# Variation at position 350 in the Chikungunya virus 6K-E1 protein determines the sensitivity of detection in a rapid E1-antigen test

**DOI:** 10.1038/s41598-018-19174-8

**Published:** 2018-01-18

**Authors:** Aekkachai Tuekprakhon, Emi E. Nakayama, Koen Bartholomeeusen, Orapim Puiprom, Tadahiro Sasaki, Ralph Huits, Natthanej Luplertlop, Nathamon Kosoltanapiwat, Pannamas Maneekan, Kevin K. Ariën, Tatsuo Shioda, Pornsawan Leaungwutiwong

**Affiliations:** 1 Department of Microbiology and Immunology, Faculty of Tropical Medicine, Mahidol University, Bangkok, Thailand; 2 Mahidol-Osaka Center for Infectious Diseases (MOCID), Faculty of Tropical Medicine, Mahidol University, Bangkok, Thailand; 3 Research Institute for Microbial Diseases (RIMD), Osaka University, Osaka, Japan; 4 Department of Biomedical Sciences, Institute of Tropical Medicine, Antwerp, Belgium; 5 Department of Clinical Sciences, Institute of Tropical Medicine, Antwerp, Belgium; 6 Department of Tropical Hygiene, Faculty of Tropical Medicine, Mahidol University, Bangkok, Thailand; 7 Department of Biomedical Sciences, University of Antwerp, Antwerp, Belgium

## Abstract

Chikungunya virus (CHIKV), a mosquito-borne pathogen, consists of three genotypes: East/Central/South African (ECSA), West African (WA), and Asian. Although a current rapid immunochromatographic (IC) test detecting CHIKV E1-antigen showed high sensitivity to ECSA-genotype viruses, it showed poor performance against the Asian-genotype virus that is spreading in the American continents. To understand the basis for the low performance of this IC test against Asian-genotype virus, we re-examined the anti-CHIKV monoclonal antibodies (mAbs) used in the assay for their interaction with E1-antigen of the three CHIKV genotypes. We found that the reactivity of one mAb for Asian-genotype virus was lower than that for ECSA virus. Comparison of E1 amino acid sequences revealed that the ECSA virus used to generate these mAbs possesses glutamic acid (E) at position 350, in contrast to WA and Asian, which possess aspartic acid (D) at this position. Site-directed mutagenesis confirmed that the mutation altered mAb reactivity, since E-to-D substitution at position 350 in ECSA reduced recognition by the mAb, while D-to-E substitution at this position in Asian and WA increased affinity for the mAb. Taken together, these results indicate that residue 350 of the CHIKV 6K-E1 is a key element affecting the performance of this IC assay.

## Introduction

Chikungunya virus (CHIKV; genus *Alphavirus*, family *Togaviridae*) is a re-emerging mosquito-borne pathogen. Three genotypes, East/Central/South African (ECSA), West African (WA), and Asian, have been classified. Genotype names reflect the primary geographical area where the respective viruses were first isolated. CHIKV was previously thought to be restricted to tropical countries. However, the range of CHIKV, along with its vectors (*Aedes* mosquitoes), continues to expand throughout the globe. Approximately 1.3 billion people world-wide live in areas at risk of CHIKV transmission^1^. Genetic adaptation of CHIKV to *Aedes albopictus*, a species infesting temperate regions, has been suggested as a main driver of CHIKV emergence in non-tropical areas^2,3^.

Although joint pain is considered as the classical hallmark of CHIKV infection, acute non-specific symptoms, affecting 75–95% of infected cases^4^, overlap with those of other viruses, such as Dengue and Zika, that thrive in the same mosquito vectors in similar geographical regions. Thus, it can be difficult for clinicians to diagnose Chikungunya solely based on clinical symptoms, especially in areas where these viruses co-circulate^5^. Therefore, laboratory testing is essential for an accurate diagnosis of CHIKV infections.

Currently, the most sensitive diagnostic methods for acute CHIKV infection still rely on intricate molecular techniques (e.g., polymerase chain reaction (PCR) -based assays) that are often unavailable in areas with limited resources. To increase the accessibility of CHIKV diagnosis, a cheap, user-friendly, rapid point-of-care diagnostic modality is needed^6,7^. To date, commercial rapid diagnostic tests for detecting CHIKV antigen are not available. However, a novel rapid immunochromatographic (IC) assay that uses monoclonal antibodies (mAbs) to detect CHIKV envelope protein E1 was developed recently by Okabayashi *et al*.^8^. CHIKV (ECSA genotype) isolated from a Thai patient was used to generate the mAbs employed in this assay^9,10^. Okabayashi *et al*. reported on the detection of the ECSA genotype in clinical samples from Thailand and Laos with a sensitivity and specificity of 89.4% and 94.4%, respectively, and in accordance with PCR results^8^. However, the results for detection of the Asian and WA genotypes were inconclusive due to the limited number of samples tested. A further clinical study showed a sensitivity for ECSA lineage detection of 88.9%, consistent with the results of Okabayashi *et al*. but a low sensitivity (33.3%) for the Asian genotype using a larger number of samples^11^.

The performance of the CHIKV antigen detection test against the Asian genotype is important, especially because the CHIKV strains that prevailed in recent epidemics in several countries in Southeast Asia and in Central and South America clustered with the Asian genotype. The introduction of the Asian genotype in the Caribbean, a popular travel destination, could enhance further intercontinental spread^12,13^. Addressing possible shortcomings of the current CHIKV antigen detection test will benefit the further rational design of rapid point-of-care CHIKV diagnostics.

There are several possible factors that affect the sensitivity of this test towards the Asian genotype that require consideration. Viral antigens are typically present in detectable amounts in blood only for the first few days after the onset of symptoms. Therefore, clinical samples from different time points following the onset of illness may exhibit differing signal levels as measured by the assay. Storage conditions may also affect native antigen levels in the samples. Additionally, the rise in immunoglobulin (Ig) levels in patient serum might interfere with the reaction of antigens with the mAbs used in the test. This phenomenon has been documented for the detection of non-structural protein 1 (NS1) of Dengue virus. The dissociation of antigen-antibody complexes has been shown to improve sensitivity in Dengue virus antigen detection assay^14,15^. Furthermore, even though the genotypes of CHIKV have been well defined, inter- and intra-genotype microevolution could occur due to genetic variation. Viral genetic variations, naturally acquired- or evolutionary forced-mutation, could subsequently lead to amino acid variation^3^. CHIKV amino acid variation has been reported to alter binding and neutralizing capacity of CHIKV-infected human sera^16^. Fluctuations in the sensitivity of serology tests for CHIKV in independent outbreaks have been postulated because of the amino acid substitution in CHIKV envelope proteins^17^. In the present study, we focus on the reactivity and binding properties of the antibodies used by Okabayashi *et al*. towards different CHIKV lineages and show that a genotype-specific amino acid substitution in E1 determines the sensitivity of antigen detection.

## Results

### Sensitivity of the rapid IC test kit to ECSA- and Asian-genotype CHIKVs

**Figure 1:**
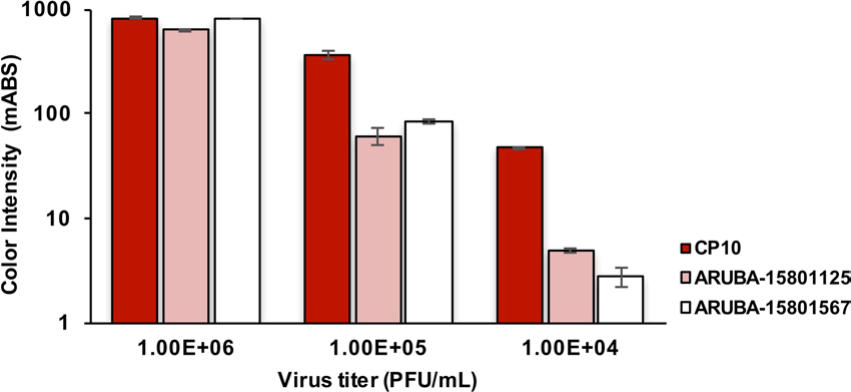
Sensitivity of the rapid immunochromatographic (IC) test kit to ECSA- and Asian-genotype chikungunya virus (CHIKV). A rapid IC kit using mouse anti-CHIKV E1 protein antibodies as tracers was tested against two different CHIKV genotypes: ECSA (CP10 strain) and Asian (ARUBA-15801125 and 15801567). The chart displays the measurement of intensity of the test line through the peak height of the immunochromatogram at three points of CHIKV titer: 1.00E + 06, 1.00E + 05, and 1.00E + 04 PFU/ml. Dark red bars represent CHIKV ECSA (CP10 strain); light red and white bars represent the ARUBA-15801125 and 15801567 strains of Asian genotype, respectively. Bar chart represents mean of intensity (milli-absorbance; mABS) and error bars indicate actual fluctuations of duplicate samples.

The clinical study by Huits *et al*.^11^ reported that the rapid IC test showed low sensitivity to specimens containing Asian genotype CHIKV. To assess possible reasons for this issue, we first conducted similar experiments using CHIKV isolates of the Asian genotype. Specifically, the assay was used to test two strains of Asian-genotype CHIKV (strains ARUBA-15801125 and 15801567 strain^11^), along with the ECSA-genotype CP10 strain. Three dilutions of each virus, at titers 1.00E + 06, 1.00E + 05 and 1.00E + 04 PFU/mL, were prepared for IC testing. All rapid IC strips gave valid results, as indicated by the presence of the control lines. For the CP10 virus, visible bands at the test line were observed in all three dilutions; in contrast, for both strains of the Asian genotype, bands were visible at the test line only in the dilutions at 1.00E + 06 and 1.00E + 05 PFU/mL. Quantitative measurement of the intensities of the test lines revealed that, at the high virus titer (1.00E + 06 PFU/mL) both the ECSA and Asian genotypes of CHIKV were detected with comparable sensitivity (Fig. 1). With 1.00E + 05 PFU/mL, the intensity of the CP10 sample was approximately 5-fold higher than that of the ARUBA-15801125 and 15801567 strains. As noted above, at the lowest titer (1.00E + 04 PFU/mL), the test line was visible by the naked eye only in CP10. At this concentration, densitometry revealed that the intensity of the band for CP10 was approximately 10-fold higher than that of the ARUBA-15801125 and 15800567 strains (Fig. 1). These results confirmed that this rapid IC test exhibited significantly decreased sensitivity to Asian-genotype viruses compared to ECSA-genotype viruses.

### Weak reactivity of CK47 to Asian-genotype CHIKV

**Figure 2:**
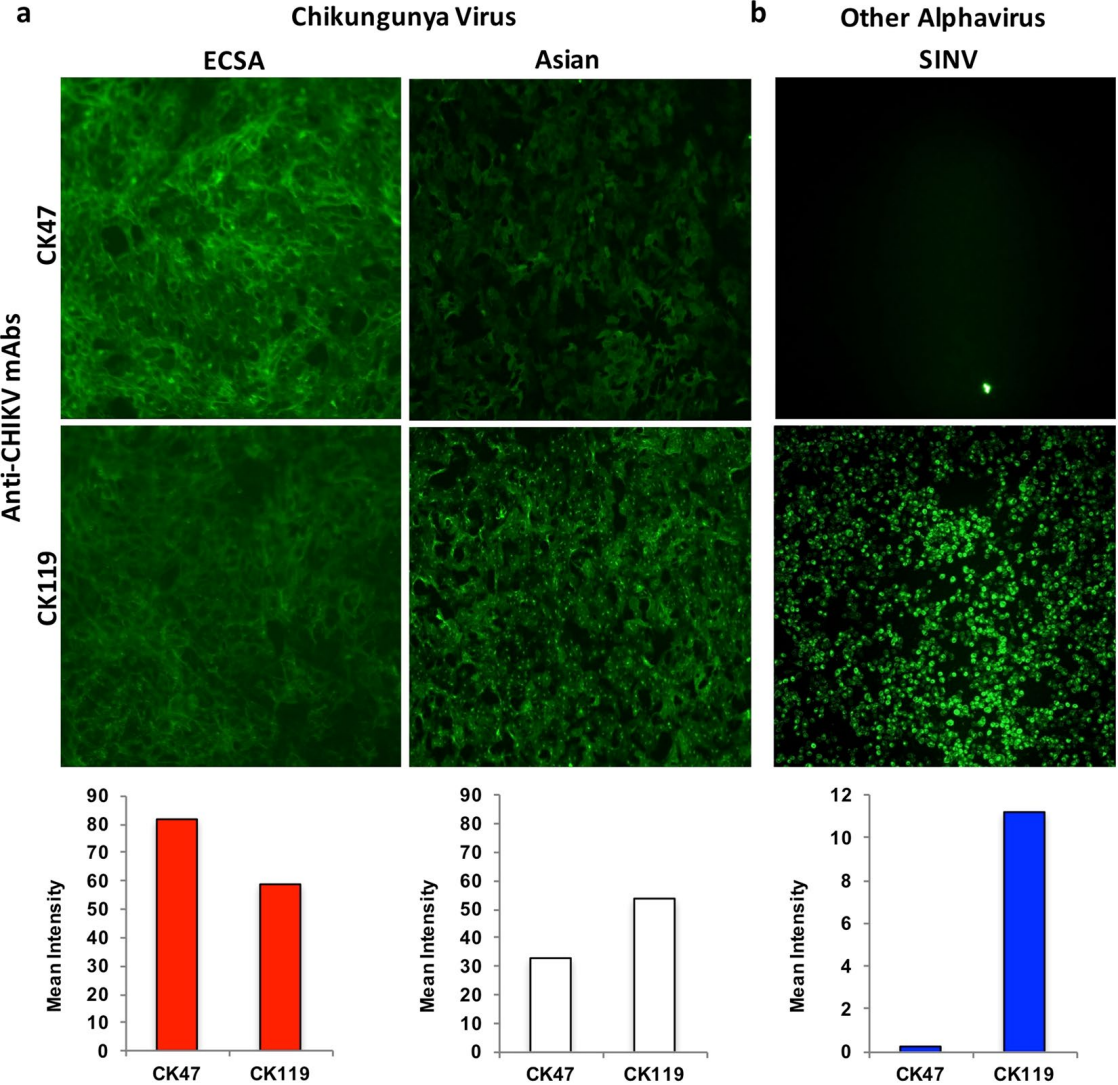
Indirect immunofluorescence analysis of CK47 and CK119 labeling of virus-infected cells. ECSA (CP10)- and Asian (ARUBA-15801125)-genotype Chikungunya virus (CHIKV) -infected Vero cells (**a**) and Sindbis virus (SINV) -infected BHK cells (**b**) were stained with CK47 or CK119; labeling was detected with Alexa Fluor 488-conjugated secondary antibody. Images are representative of results obtained from two independent experiments and were taken under 10 X objective magnification. Alexa Fluor 488 signals of each photo were quantified by NIS-Elements and were expressed as a mean intensity per pixel. Red, white, and blue bars are signals of ECSA-CHIKV, Asian-CHIKV, and SINV, respectively.

To evaluate the binding specificity of the anti-CHIKV mAb used in the CHIKV E1 antigen rapid IC test, we performed immunofluorescence analysis of the activity of purified CK47 and CK119 against Vero cells infected with CHIKV of the ECSA (CP10 strain) or Asian (ARUBA-15801125 strain) genotype. Reactivity of both mAbs to CHIKV-infected Vero cells is shown in Fig. 2a and Supplementary Fig. S1. For the ECSA genotype, CK47 reacted with CP10-infected Vero cells better than CK119 did. In contrast, the reactivity of CK47 to ARUBA-15801125-infected cells was significantly weaker than that of CK119. Six other strains of the Asian genotype (ARUBA-15801136, 15801160, 15801358, 15800567, 15802650, and 15801654) were tested with CK47 and CK119, with similar results to that seen with ARUBA-15801125 (data not shown). As a control, CK47 and CK119 were tested against the closely related Sindbis virus (SINV; *Togaviridae*) (Fig. 2b and Supplementary Fig. S1). CK47 did not bind SINV-infected BHK cells, confirming the highly specific reactivity of CK47 to CHIKV, as reported previously by Okabayashi *et al*.^8^. In contrast, CK119 showed strong cross reactivity with SINV-infected cells (Fig. 2b and Supplementary Fig. S1). This result raised another concern regarding the use of CK119 as a component of the E1 CHIKV rapid detection test, although (notably) this assay does not detect cultured SINV^8^.

### Amino acid variation at position 350 of the CHIKV 6K-E1 protein

**Figure 3:**
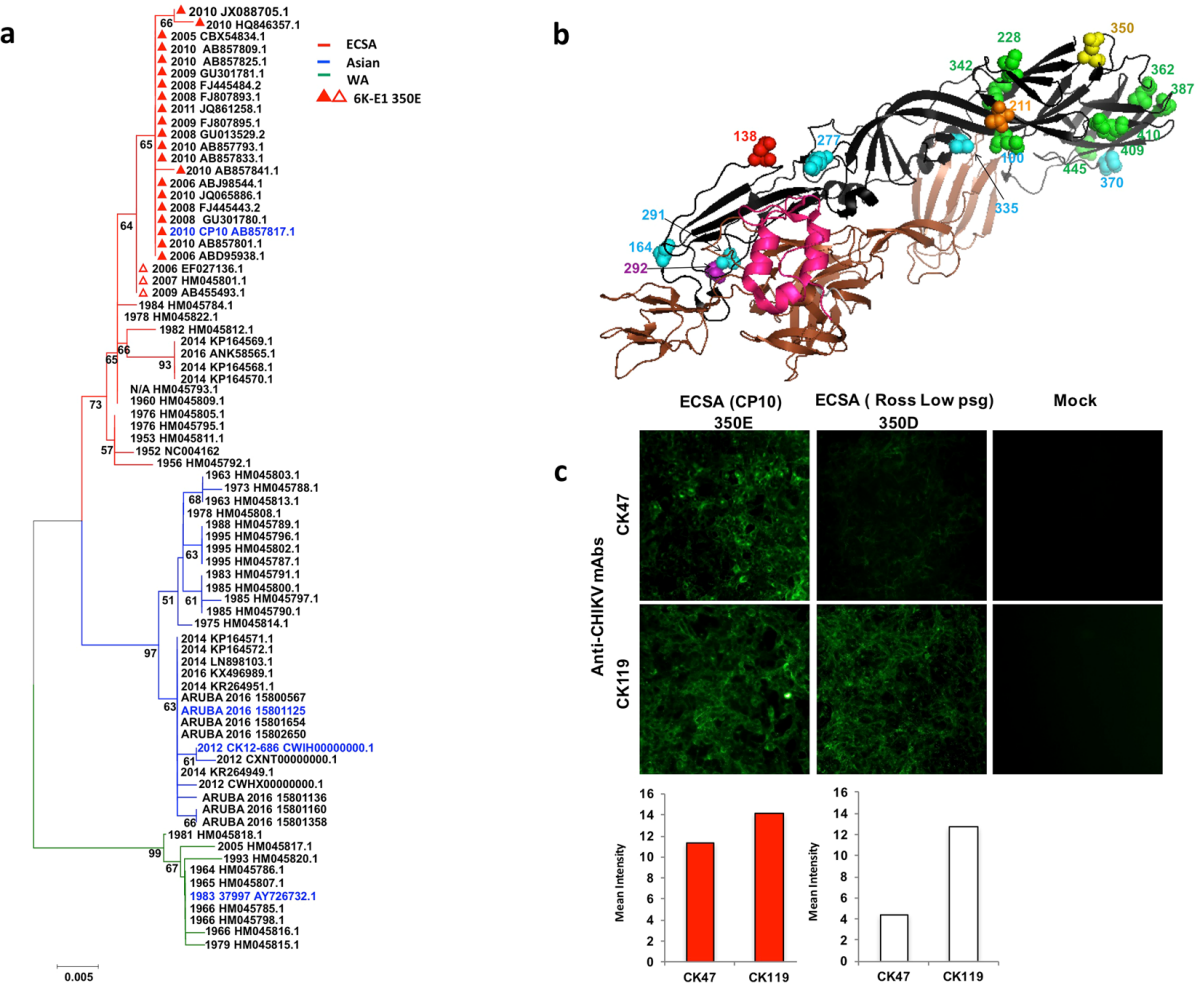
Amino acid variation at position 350 of the CHIKV 6K-E1 protein. (**a**) Nearest-neighbor-joining tree with 1,000 bootstraps of sequences from the indicated CHIKV strains was constructed using MEGA version 7 (Kumar, Stecher, and Tamura 2015). Scale bar denotes the genetic distance; numbers below the branches represent percentage of associated taxa clustered together. The three major genotype branches are shown in different colors: ECSA (red), Asian (blue), and WA (green). Collection year and Genbank accession number of each sequence is shown. Red triangles indicate CHIKV strains with glutamic acid (E) at position 350 of the 6K-E1 protein. Among those strains, closed or open triangles indicate valine (V) or alanine (A) (respectively) at amino acid position 292. CHIKV strains without a triangle possess aspartic acid (D) and “A” at positions 350 and 292, respectively. CHIKV strains used in plasmid construction and ARUBA-15801125 are indicated by blue. (**b**) 3D-structure of CHIKV glycoproteins based on Protein Data Bank number 3N42 using The PyMOL Molecular Graphics System, Version 1.8 Schrödinger, LLC. E3 is shown in magenta, E2 in brown, and E1 in black. Spheres on E1 indicate amino acid positions that vary among the three genotypes of CHIKV. Purple and yellow spheres indicate amino acids at position 292 and 350, respectively. Red, green, and light-blue spheres indicate the ECSA-, WA- and Asian-genotype-specific amino acid residues, respectively. Orange spheres indicate amino acid positions that differ among all three genotypes. (**c**) Indirect immunofluorescence test of mAb labeling of CHIKV-infected cells. CP10 (350E), Ross Low psg strain (350D), or mock-infected Vero cells were stained with CK47 and CK119; labeling was detected with Alexa Fluor 488-conjugated secondary antibody. Images are representative of results obtained from two independent experiments and were taken under 10X objective magnification. Red and white bars are fluorescent signal levels of 350E and 350D, respectively.

Previously, it was reported that CK47 recognized the CHIKV E1 protein^10^. From the NCBI database, we retrieved 77 sequences of predicted CHIKV 6K-E1 proteins, representing the three CHIKV genotypes during various major outbreaks around the world. The sequences were subjected to multiple sequence alignment to explore amino acid variation among genotypes. The 6K-E1 protein from the prototypical CHIKV S27 strain, isolated in Tanzania in 1952, was used as the reference amino acid sequence. Details of amino acid variation of selected strains are shown in Table 1. A phylogenetic tree was constructed to show the evolutionary relationship among the strains. Strains sorted into three major branches or groups, corresponding to the major CHIKV genotypes (Fig. 3a and Supplementary Table S1). In total, we observed 19 positions within E1 that displayed amino acid variations among strains of different genotypes (Fig. 3b).

**Table 1:** Amino acid variation in CHIKV E1 proteins.

Country	Year	Strain	Accession No.	Genotype	Amino acid position																		
					34	72	98	145	162	211	225	226	269	276	284	296	304	321	343	344	379	404	420*
					100	138	164	211	228	277	291	292	335	342	350	362	370	387	409	410	445	470	486**
TANZANIA	1952	S27	NC004162	Prototype	L	N	A	T	I	K	A	A	M	M	D	L	P	A	E	I	E	A	V
TANZANIA	1953	Ross Low psg	HM045811.1	ECSA	.	.	.	.	.	.	.	.	.	.	.	.	.	.	.	.	.	.	.
SRILANKA	2009	SL111314	AB455493.1	ECSA	.	.	.	.	.	.	.	.	V	.	E	.	.	.	.	.	.	.	.
MALAYSIA	2009	0901aTw	FJ807895.1	ECSA-IOL	.	.	.	.	.	.	.	V	V	.	E	.	.	.	.	.	.	.	.
THAILAND	2010	CP10	AB857817.1	ECSA-IOL	.	.	.	.	.	.	.	V	V	.	E	.	.	.	.	.	.	.	.
CAMBODIA	2011	V1024314 KH11 PVH	JQ861258.1	ECSA-IOL	.	.	.	.	.	.	.	V	V	.	E	.	.	.	.	.	.	.	.
BRAZIL	2016	BR33	KX228391.1	ECSA	.	.	.	.	.	.	.	.	V	.	.	.	.	.	.	.	.	.	.
SENEGAL	1983	37997	AY726732.1	WA	Q	S	.	A	V	.	.	.	V	I	.	V	.	T	D	V	A	T	I
SENEGAL	2005	HD 180760	HM045817.1	WA	Q	S	.	A	V	.	.	.	I	I	.	V	.	T	D	V	A	T	I
THAILAND	1975	1455–75	HM045814.1	Asian	.	S	T	S	.	E	S	.	.	.	.	.	S	.	.	.	.	.	.
INDONESIA	1983	JKT23574	HM045791.1	Asian	.	S	T	S	.	E	S	.	.	.	.	.	S	.	.	.	.	.	.
PHILIPPINES	2012	CK12–686	CWIH00000.1	Asian	.	S	T	A	.	E	S	.	.	.	.	.	S	.	.	.	.	.	.
ARUBA***	2016	ARUBA-15801125	MF682981	Asian	.	S	T	A	.	E	S	.	.	.	.	.	S	.	.	.	.	.	.

*Amino acid position in E1 protein.

**Amino acid position in 6K-E1 protein.

***Aruba isolates, ARUBA-15801136 (MF682982), 15801160 (MF682983), 15801358 (MF682984), and 15801654 (MF682985), possess the same amino acid residues as ARUBA-15801125 at positions shown in this table.

Among these 19 positions, we focused on position 350 of the 6K-E1 protein (corresponding to position 284 of the E1 protein, see Table 1), since a glutamic acid (E) to-glycine (G) substitution at this position was previously reported in a variant CHIKV that had escaped from an inhibitory effect by CK47^10^. We found that clinical isolates of CHIKV strains possessed either of two amino acids at this position: aspartic acid (D) or E. All of the Asian or WA genotype sequences harbored a D at position 350 (350D). However, all strains of the ECSA genotype isolated during the large outbreaks of 2005–2011 possessed E at position 350 (350E), which distinguished these isolates from other ECSA-genotype viruses encoding D at this position. This result was confirmed by phylogenetic analysis, where strains of the ECSA genotype with 350E were found to stem from the same common ancestor (Fig. 3a and Supplementary Table S1). Most ECSA strains with 350E were classified as members of the Indian Ocean Lineage (IOL). Given that CK47 was derived from a mouse immunized with an ECSA-genotype virus encoding the 350E envelope protein, our results may explain the restricted specificity of CK47, which appears to preferentially detect viruses of the ECSA genotype with 350E-containing envelope proteins.

To confirm this finding, we also performed Indirect Immuno Fluorescence Test (IIFT) for CK47 against ECSA-genotype viruses encoding 350D (Ross Low psg strain) and 350E (CP10 strain). As expected, the reactivity of CK47 to CP10 was significantly higher than that to Ross Low psg, even though both viruses are ECSA genotype (Fig. 3c and Supplementary Fig. S1).

### Pivotal role of amino acid 350 in the 6K-E1 protein for CHIKV antigen:CK47 interaction

**Figure 4:**
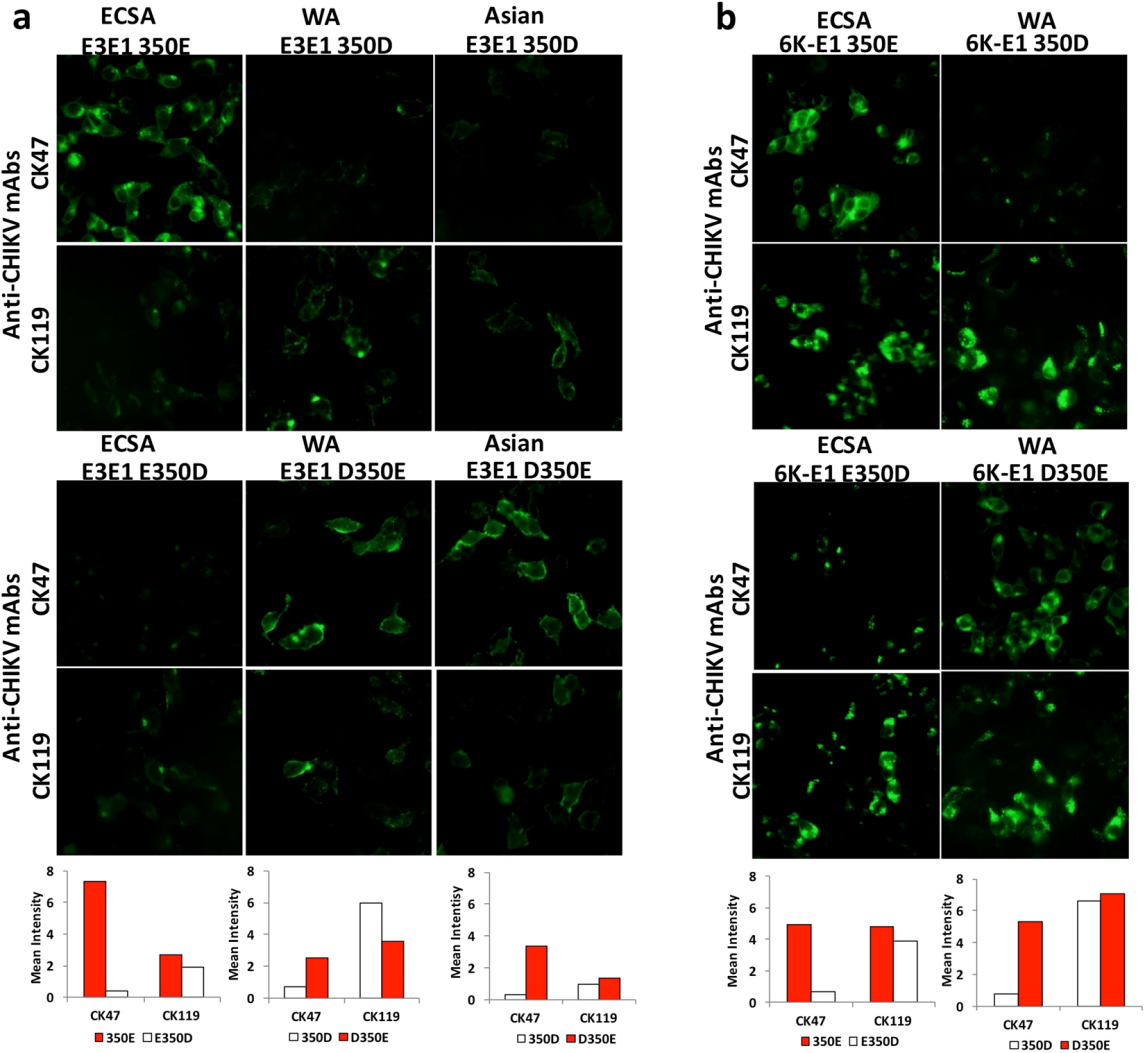
Amino acid at position 350 is a key element of CHIKV antigen:CK47 interaction. Plasmids encoding CHIKV envelope protein from each of the three genotypes were transfected into HEK293T cells and Indirect Immunofluorescence test was performed using CK47 and CK119; labeling was detected with Alexa Fluor 488-conjugated secondary antibody. Plasmids were constructed to encode the full-length CHIKV envelope protein (E3-E2–6K-E1) from the CP10 (ECSA), 37997 (WA), or CK12–686 (Asian) strain, or mutant envelope proteins from CP10 bearing E350D, 37997 bearing D350E, or CK12–686 bearing D350E (**a**). Plasmids were constructed to encode the short versions of the envelope protein (6K-E1) from the CP10 strain (ECSA) or 37997 (WA) strain bearing either the wild type or mutant residue at position 350 (**b**). Images are representative of results obtained from three independent experiments and were taken under 20X objective magnification. Red and white bars below photos are fluorescent signal levels of 350E and 350D versions of the indicated envelope proteins, respectively.

To prove the effect of amino acid variation at position 350 in the 6K-E1 protein on CK47 binding, we constructed plasmids expressing the CHIKV envelope proteins (E3-E2–6K-E1) from each of the three genotypes. The CHIKV CP10, 37997, and CK12–686 strains (indicated by blue colors in Fig. 3a) were used as representatives of viruses of the ECSA, WA, and Asian genotypes, respectively. We introduced an E-to-D mutation at position 350 of the CP10 protein, and D-to-E mutations at the same position of the 37997 and CK12–686 proteins, resulting in plasmids ECSA E350D, WA D350E, and Asian D350E, respectively. The parent and mutated plasmids were transfected into HEK293T cells and IIFT was performed to assess reactivity of CK47 to each of the corresponding envelope proteins. CK119 staining was used as the expression control for each of the transfected cell lines. Among cells expressing the respective wild-type protein (Fig. 4a and Supplementary Fig. S2), cells expressing the 350E-containing ECSA envelope protein exhibited the strongest reactivity to CK47. The introduction of the E350D mutation significantly reduced the reactivity of CK47 against cells expressing the ECSA protein, while the introduction of the D350E mutation significantly increased the reactivity of CK47 against cells expressing the WA- or Asian-derived proteins (Fig. 4a and Supplementary Fig. S2). In contrast, CK119 showed no difference in reactivity against cells expressing proteins containing either 350E or 350D.

To exclude possible interference by other positions in E3-E2, we constructed plasmids expressing the respective wild-type and mutant 6K-E1 proteins possessing E or D at position 350. Consistent with the above results, CK47 displayed stronger reactivity to proteins containing 350E than to those harboring 350D, regardless of the E1 backbone being ECSA or WA genotype (Fig. 4b and Supplementary Fig. S2). Taken together, these results clearly prove that the amino acid at position 350 of the 6K-E1 protein plays a key role in the antigen-antibody interaction between CK47 and the CHIKV E1 protein.

### No effect of substitution at residue 292 in 6K-E1 on reactivity with CK47

**Figure 5:**
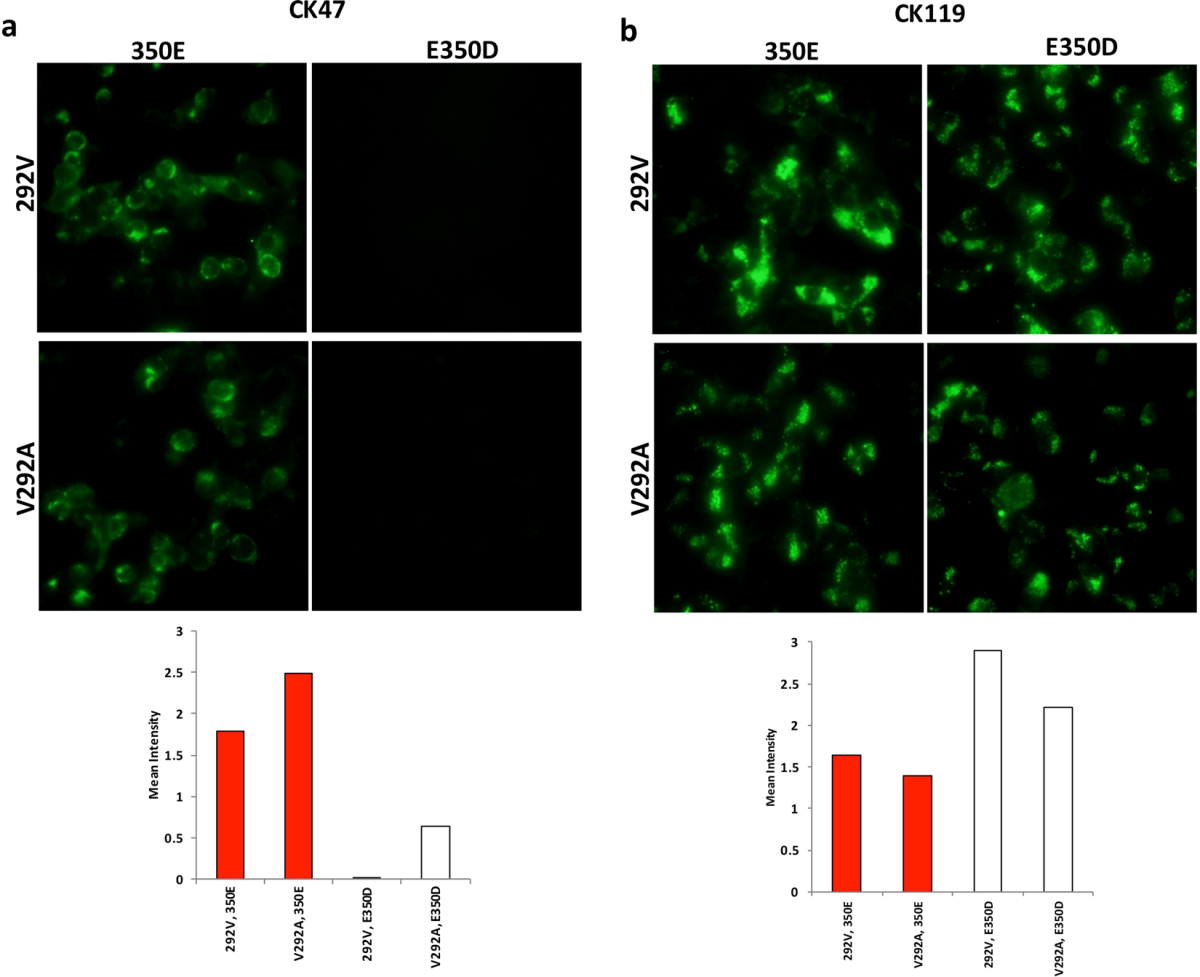
Effect of amino acid substitution at residue 292 of 6K-E1 on reactivity with CK47. Site-directed mutagenesis was used to introduce mutations encoding substitutions at positions encoding residues 292 and 350 of 6K-E1 from the CP10 strain. Four possible combinations of 292 and 350 amino acid positions were produced: 292 V + 350E, 292 V + E350D, V292A + 350E, and V292A + E350D. Immunofluorescent images display reactivity of CK47 (**a**) and CK119 (**b**) against proteins of each of these four sequences. Images are representative of results obtained from two independent experiments and were taken under 20X objective magnification. Red and white bars below photos are fluorescent signal levels of 350E and 350D versions of the indicated envelope proteins, respectively.

After the La Reunion outbreak in 2005, microevolution of CHIKV was noted in the ECSA genotype. Notably, an alanine (A) –to-valine (V) substitution at position 226 (A226V) of E1 was reported to affect CHIKV adaptation to *Aedes albopictus*^3^. In our multiple sequence alignment, besides the variation in position 350 of 6K-E1, we also observed a pattern of amino acid changes at position 292 of 6K-E1, a residue that corresponds to position 226 of E1. We found three patterns of amino acid variations at positions 292 and 350 of 6K-E1, as shown in Table 1. The prototype ECSA, Asian, and WA strains encode 292A and 350D, whereas the ECSA-IOL encodes 292 V and 350E (marked with closed red triangles in Fig. 3a). Interestingly, some ECSA strains encode 292A and 350E (marked with open triangles in Fig. 3a); we infer that these isolates were intermediate strains undergoing initial changes. Based on these data, we then explored the possible effects of these amino acid combination patterns on the CK47:E1 interaction. Site-directed mutagenesis was done to construct plasmids encoding the 6K-E1 protein of CP10 with each of the four possible combinations of amino acids at positions 292 and 350. We observed that the reactivity of CK47 was only affected when 350D was present (Fig. 5 and Supplementary Fig. S3) and not with amino acid variation at position 292.

### Potent inhibition of CHIKV envelope-pseudotyped lentivirus infection by CK47

**Figure 6:**
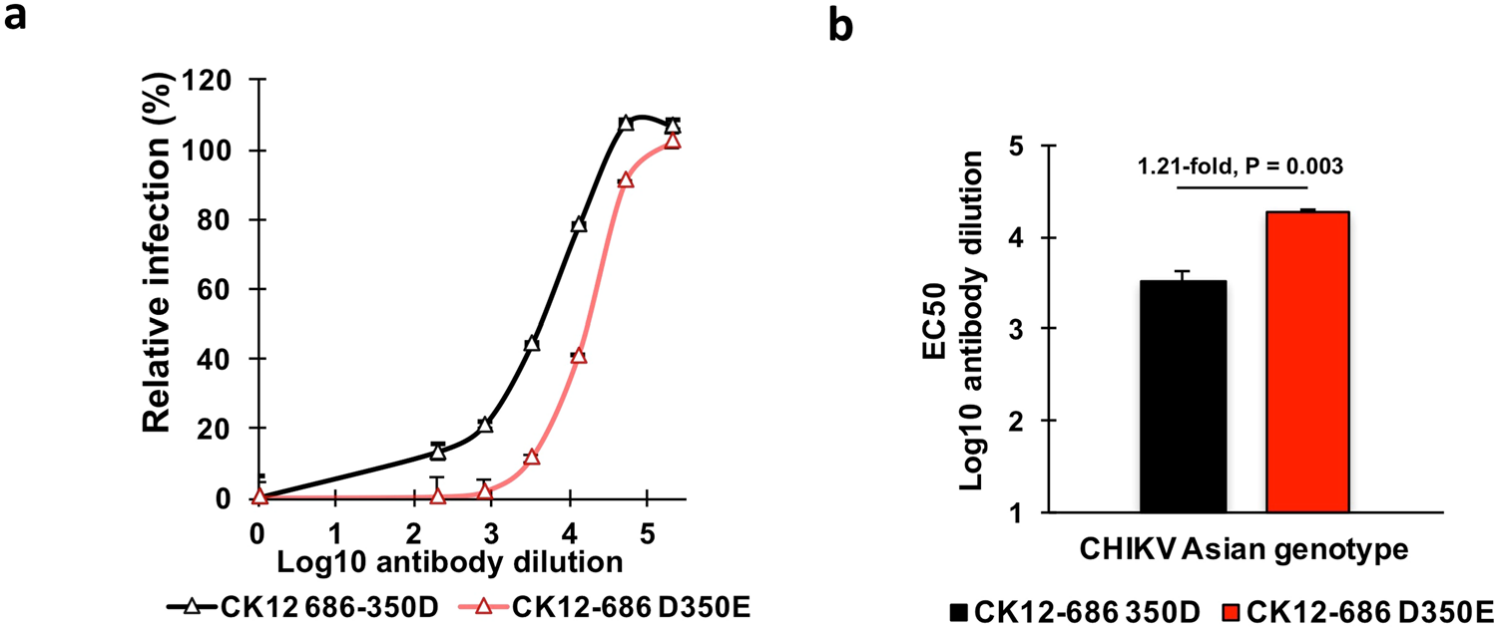
CK47 neutralization of Asian-genotype CHIKV envelope-pseudotyped lentiviral infection. Asian-genotype CHIKV (CK12–686 strain) -pseudotyped lentiviruses harboring a luciferase-encoding reporter gene were generated by plasmid transfection. Two different types of viruses were produced: wild type (encoding aspartic acid at position 350; 350D) and mutant (mutated to encode glutamic acid at position 350; D350E). Prior to infection of cells, pseudotyped lentiviruses were pre-incubated for two hours with four-fold serial dilutions of CK47 (ranging from 1:200 to 1:204800). Infectivity was evaluated at 72 hours post-infection by measuring luciferase activity. (**a**) Dose-dependent inhibition curve of CHIKV-pseudotyped lentivirus by CK47; CHIKV Asian genotype wild type (CK12–686 350D) is shown as a black line and mutant (CK12–686 D350E) is shown as a red line. Data represents mean of infection normalized to pseudotyped lentivirus-infected cells without antibody; error bars indicate standard error of the mean (SEM) of three independent experiments. (**b**) Probit analysis was performed to determine value of the mAb dilution that showed inhibition of half of the maximum infection (EC_50_) by Asian-genotype virus. Bars represent mean EC_50_ of CK47 on CHIKV Asian genotype wild type CK12–686 350D (black), and mutant CK12–686 D350E (red); error bars indicate SEM. P-values indicate statistical significance of mean EC_50_ analyzed by unpaired, two-tailed Student *t*-test.

The above results demonstrate that CK47 binds the envelope protein with lower avidity when the 6K-E1 protein harbors D rather than E at amino acid position 350. We further explored the neutralizing activity of CK47 using Asian-genotype CHIKV as a model. CHIKV-pseudotyped lentiviral particles harboring a luciferase reporter and bearing E3-E2–6K-E1 CHIKV envelope proteins were produced from CK12–686, a strain that encodes the 350D protein as the native envelope. The D-to-E amino acid substitution at this position was introduced to construct a CK12–686 mutant-pseudotyped lentivirus. Four-fold serial dilutions of CK47 (ranging from 1:200 to 1:204800) were mixed with both wild-type-and mutant-pseudotyped lentiviral particles prior to infection of U251 cells. The resulting dose-dependent inhibition curves (Fig. 6a) revealed the neutralizing activity of CK47 against CHIKV envelope-pseudotyped lentiviral particles. Notably, CK47 showed decreased neutralization activity against wild-type (350D) -pseudotyped lentivirus compared to the mutant (350E) -pseudotyped lentivirus.

The antibody dilutions calculated to yield inhibition of 50% of maximum infection (EC_50_) are shown in Fig. 6b. The EC_50_ values in wild-type- and mutant-pseudotyped viruses were 3.52 and 4.26 log_10_ antibody dilutions, respectively. Thus, wild-type-pseudotyped lentivirus showed significantly lower sensitivity to CK47 than the mutant (350E) (1.21-fold, *P* = 0.003). This result was consistent with the binding activity results described above that CK47 exhibit greater ability to interact with CHIKV harboring E at position 350 of the 6K-E1 protein.

### CHIKV 6K-E1 amino acid 350 determines sensitivity of the rapid IC test

**Figure 7:**
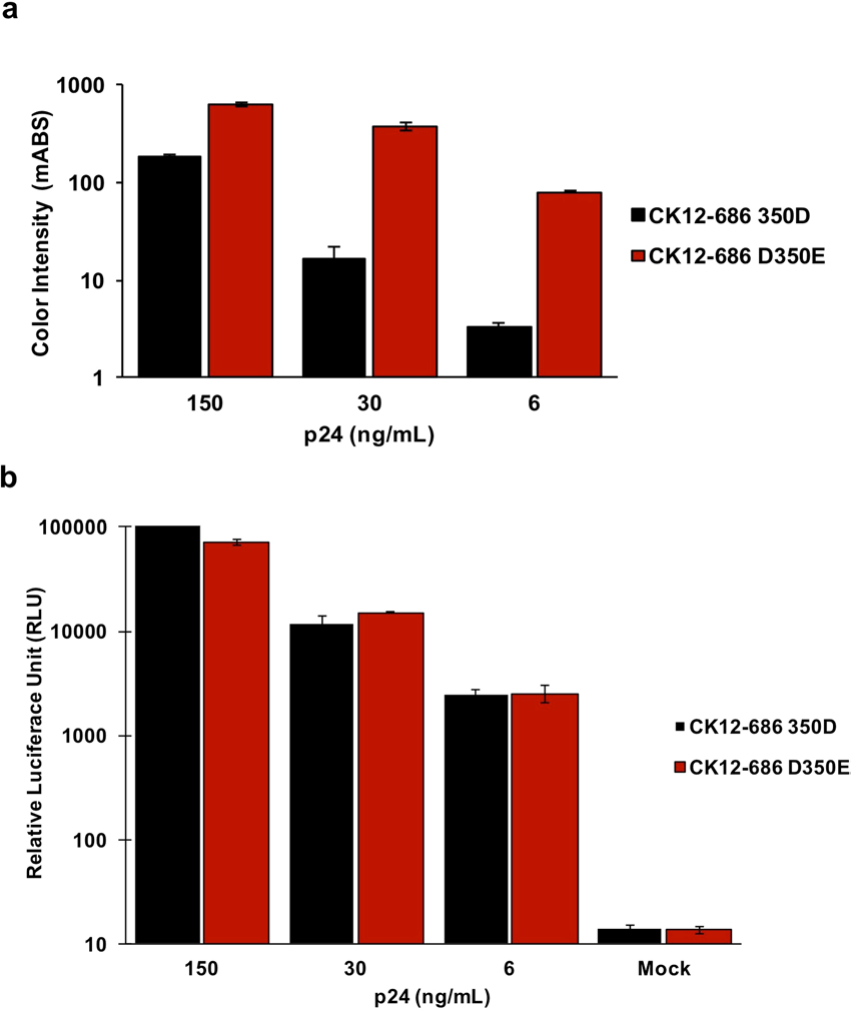
Variation of amino acid 350 of envelope 1 protein has a pivotal effect on the performance of the rapid immunochromatographic (IC) test against Asian-genotype CHIKV. A rapid IC test, using CK47 and CK119 as tracers, was performed against pseudotyped lentiviruses bearing either of two CHIKV envelope proteins: Asian-genotype strain CK12–686 wild type (350D) and mutant (D350E). Both pseudotyped lentiviruses were prepared at each of three concentrations of lentiviral capsid protein (150, 30, and 6 ng/mL). (**a**) Measurement of intensity of the test lines. Black and red bars indicate wild-type- and mutant-pseudotyped lentiviruses (respectively) bearing CHIKV envelope protein of the Asian genotype. Bars represent mean of intensity (milli-absorbance; mABS); error bars indicate actual fluctuations of duplicate samples. (**b**) Infectivity of pseudotyped lentiviruses bearing either of two CHIKV envelope protein: Asian genotype strain CK12–686 wild type and mutant. HEK293T cells were infected with the same concentrations of wild-type- and mutant-pseudotyped lentiviruses as used in kit evaluation. Infectivity was evaluated at 72 h post-infection by measuring luciferase activity as relative luciferase units (RLU). Bars represent mean of RLU of wild type (black) and mutant (red); error bars indicate standard error of the mean (SEM).

Given our demonstration that amino acid 350 in 6K-E1 is central in the interaction with CK47, we inferred that the amino acid change at this position is the cause of the lower sensitivity of the rapid IC assay to Asian-genotype CHIKV. To further prove this point, we tested the rapid IC assay against pseudotyped lentiviruses bearing the CHIKV envelope protein of the Asian genotype (strain CK12–686). Separate preparations pseudotyped with either the wild type (350D) or mutant (350E) 6K-E1 protein were generated. Amount of input virus was adjusted based on capsid protein concentration (150, 30, or 6 ng/mL). All the rapid IC strips gave valid results, as shown by the presence of the control lines. In the case of wild type virus (350D), the test line could be visualized by naked eye, but only at the highest concentration (150 ng/mL). Consistent with our hypothesis, the reactive bands at the test line of the mutant virus (350E) were observed at all concentrations, with the line intensity showing concentration dependence. Quantitative measurement of test line intensities (Fig. 7a) revealed that the intensities with the 350E virus were 3.5-fold, 24-fold, and 24-fold higher than those obtained with the 350D virus at the respective concentrations of 150, 30, and 6 ng capsid protein/mL. Furthermore, the wild type (350D) and mutant (350E) viruses yielded similar luciferase activities upon infection (Fig. 7b), excluding the possibility of increased envelope protein incorporation as a result of the mutation. These results thus proved that variation at amino acid position 350 in 6K-E1 is a pivotal factor affecting the performance of rapid IC test against Asian-genotype CHIKV.

## Discussion

A novel rapid IC test targeting the CHIKV E1-antigen has shown promising performance against specimens harboring ECSA-genotype CHIKV^8^. However, this test has shown low sensitivity against specimens containing Asian-genotype CHIKV^11^. In the present study, we explored possible reasons for the decreased sensitivity against Asian-genotype. Our results revealed that one of the mAbs used in the rapid IC assay has significantly lower reactivity against Asian-genotype. The plasmid transfection assays and IIFT demonstrated that amino acid position 350 of 6K-E1 (corresponding to position 284 of E1) is a key factor affecting the binding of CK47.

ECSA CHIKVs in the IOL encode E at this position, while Asian, WA, and remaining ECSA CHIKVs encode D at this position. E and D are chemically similar and are often exposed at the surface of proteins due to the hydrophilic properties of these residues. A study by Lee *et al*. indicated that a D-to-E substitution increases the conformational entropy of a protein^18^. Such an effect could be a plausible explanation for the restricted capacity of CK47 to bind to E1 protein with a D residue. There are two possible scenarios for this phenomenon. First, position 350 on 6K-E1 is not the major CK47 binding site but the E-to-D substitution here caused a conformational change in the epitope recognized by CK47, thus impeding the ability of CK47 to bind. Second, it is possible that position 350 on 6K-E1 is the major CK47 binding site. Although further studies on epitope structure are required to reach a definite conclusion, the second explanation may be more likely, since CK47 has proven to bind to both conformational and linear epitopes^10^.

During the outbreak of ECSA genotype CHIKV in La Reunion, adaptation of the causative CHIKV strain to the endemic vector species *Aedes* *albopictus* had been observed. This adaptation was attributed to a single amino acid mutation at residue 226 of the CHIKV E1 protein^3,19^. In our sequence alignment, we found 19 substitutions among the E1 proteins of various isolates, with changes at position 292 of 6K-E1 (corresponding to residue 226 of E1) appearing to be in strong linkage disequilibrium with position 350 of 6K-E1. Three patterns were found (292 A + 350D, 292 A + 350E and 292 V + 350E), presumably reflecting the order of evolution. The D-to-E mutation at position 350 was more likely to emerge first, followed by the A-to-V mutation at position 292. Nevertheless, we found no effect (in our assays) of the A292V substitution on reactivity with CK47, indicating that the amino acid residue at position 350 is the major determinant of CK47 binding. At present, the effect of other mutations in 6K-E1 on CK47 binding cannot be excluded, and further studies will be required to define the precise interaction between the CHIKV E1 protein and the CK47 antibody. It would also be interesting to investigate whether or not amino acid substitution at position 350 affects growth in *Aedes* *albopictus*.

Although several mouse mAbs with activity against CHIKV E1 protein have been described^10,20^, the E1 protein is known to be less immunogenic than the E2 protein in human infection cases^21,22^. Additional analyses of the CHIKV strains in Table 1 showed that inter-genotype variation of E1 is consistently smaller than that of E2. Specifically, we found that, although of similar size, a larger number of positions had mutated in the E2 proteins (32 out of 417; 7.7%) than in the E1 proteins (19 out of 439; 4.3%). Nevertheless, it will also be interesting to investigate whether or not the D-to-E substitution in ECSA IOL was driven by host immunity of infected individuals against CHIKV. Detailed serological analysis by using anti-CHIKV-IgG-positive human sera is now underway.

Together, our results indicate that a single amino acid substitution at position 350 of 6K-E1 is the key factor affecting the CHIKV E1-antigen detection by mAb CK47. Therefore, we emphasize the importance of viral mutation analysis for immunodiagnostic assay development. The generation of a new panel of broadly reacting mAbs for CHIKV diagnosis is clearly a priority, and is currently under way.

## Materials and Methods

### CHIKV-specific mouse mAbs

Antibodies CK47 and CK119^10^ were purified from the ascites fluids of mice injected with the respective hybridoma cells. To ensure consistency throughout the study, each of the two antibodies was produced as a single batch. The mouse experiments for the preparation of mAbs were approved by the ethics commitee of Mahidol University, Thailand. All the mouse experiments were performed in accordance with the guidelines and regulations of Mahidol University.

### Immunochromatographic (IC) test

The details of the kit components have been described previously^8^. Briefly, two mouse anti-CHIKV mAbs were used in the rapid IC kit: CK47, which functions as a CHIKV antigen-capture mAb, was immobilized onto the membrane at the test line; CK119, provided as a gold nanoparticle-conjugated-mAb, was placed at the sample pad. Thirty microliters of serially diluted culture supernatant containing CHIKV or CHIKV-pseudotyped lentiviral vector at the indicated concentration were mixed with 30 µl IC kit extraction buffer in a tube. The IC stick then was inserted into the tube of diluted supernatant to start the reaction. After 15 min, the appearance of the control and test lines was assessed. An IC Reader C10066–10 (Hamamatsu Photonics K.K., Japan) was also used to quantitate the intensities of the test lines; values were expressed as milli-absorbance units (mABS).

### Cells and viruses

Two of the cell lines, African green monkey kidney epithelium cells Vero (ATCC CCL-81) and a glioblastoma-derived human cell line U251 (JCRB IFO50288), were maintained in Minimum Essential Medium (MEM; Life Technologies, Inc.). Human Embryonic Kidney cells HEK293T (ATCC CRL-3216) and Baby Hamster Kidney fibroblast cells BHK (ATCC CCL-10) were maintained in Dulbecco’s Modified Eagle Medium (DMEM; Life Technologies). For standard cultivation, the indicated medium was supplemented with 10% (v/v) heat-inactivated Fetal Bovine Serum (FBS; Life Technologies); for infected cells, medium was supplemented to only 2% (v/v) FBS. All cells were maintained at 37 °C in a 5% CO_2_ environment.

Two genotypes of CHIKV were used in this study. The ECSA genotype was represented by strain CP10, which was originally isolated during a 2010 outbreak in Thailand^9,23^. The Asian genotype was represented by seven strains (ARUBA-15801125, 15801136, 15801160, 15801358, 15801567, 15801650, and 15801654) that were originally isolated from the serum of patients in Aruba^11^. Sindbis virus (SINV; R68 strain), another alphavirus, was kindly provided by Dr. Kohji Moriishi, University of Yamanashi. CHIKV strains were propagated using Vero cells; SINV was propagated using BHK cells. For viral titration, a standard plaque assay was performed^24^.

### Expression vectors and site-directed mutagenesis of the CHIKV E1-encoding genes

To study the reactivity of mAbs against native and mutated CHIKV envelope proteins, we employed a plasmid-based system. Codon-optimized complementary DNAs (cDNAs) encoding (separately) the E3-E2–6K-E1 and 6K-E1 CHIKV envelope proteins were synthesized by a commercial vendor (GenScript, Piscataway, NJ). The gene sequences were designed to encode envelope proteins corresponding to those of the CP10 (GenBank accession number AB857817.1), 37997 (AY726732.1), and CK12–686 (CWIH01000001.1) strains as representatives of the ECSA, WA, and Asian genotypes, respectively. These cDNAs were subcloned into the pCAGGS MSII mammalian expression plasmid. Site-directed mutagenesis was performed to generate versions encoding mutated 6K-E1 proteins, including a valine-to-alanine substitution at position 292 (V292A) and an aspartic acid-to-glutamic acid substitution at position 350 (D350E) in the CP10-strain gene, and E350D substitutions in the 37997- and CK12–686-strain genes. Site-directed mutagenesis was performed using the overlapping primer method as described previously^25^.

### CHIKV-pseudotyped lentiviral vector

A safe, highly efficient CHIKV-pseudotyped Human Immunodeficiency Virus type 1 (HIV-1) -derived lentiviral vector was used to deliver CHIKV envelope protein on virus particles. The production of the CHIKV-pseudotyped lentiviral vector was as described previously^26^ with some modifications. Briefly, three essential plasmids were used. The first plasmid was a pCAGGS MSII plasmid harboring a DNA cassette encoding the Asian-genotype CHIKV E3-E2–6K-E1 protein, corresponding to the CK12–686 strain. The second plasmid was pLenti CMV Puro LUC (w168–1) carrying a reporter gene encoding firefly luciferase (Addgene, Cambridge, MA). The third plasmid consisted of the psPAX2 lentivirus packaging vector (Addgene). These plasmids were added to 1 mL of plain DMEM medium at a ratio of 2.6 µg: 9.0 µg: 9.0 µg (respectively); this volume was then supplemented with 40 µL polyethelenimine (Polysciences, Inc., Warrington, PA). The mixture was then added to a monolayer of HEK293T cells. After 6 h of transfection, the culture medium was replaced with fresh DMEM containing 10% FBS, and cultivation was continued for another 48 h. For each lot of lentiviral vector, titer was detected by measuring the level of HIV-1 Gag p24 protein using the RETROtek HIV-1 p24 Antigen-ELISA kit (Zeptometrix; Buffalo, NY), according to manufacturer’s instructions.

### Indirect ImmunoFluorescence Test (IIFT)

To determine the reactivity and cross reactivity of CHIKV-specific mAbs, IIFT was performed. We prepared two kinds of 96-well plate cultures for IIFT, using either live-virus-infected cells or plasmid-transfected cells. CHIKV or SINV were added to monolayers of Vero or BHK cells, respectively, at a multiplicity of infection (MOI) of 0.1. Infected cells were maintained until cytopathic effects were observed. Infected cells then were fixed with 3.7% (v/v) formaldehyde-containing phosphate-buffered saline (PBS) for 20 min, permeabilized with 1% Triton X-100 for 5 min, and washed with PBS. For plasmid-transfected cells, pCAGGS MSII expressing CHIKV envelope proteins was transfected to HEK293T cells at 80–90% confluence by Lipofectamine 2000^®^ (Invitrogen, Carlsbad, CA). Transfected cells were incubated at 37 °C in a 5% CO_2_ environment. After 48 h, transfected cells were fixed, permeabilized, and washed as above.

Primary antibody (purified anti-CHIKV mAb at 2 µg/mL) was dispensed at 50 µL/well into 96-well plates containing fixed infected or transfected cells. After incubation for 1 h at 37 °C, unbound antibody was removed by aspiration following by washing three times with 100 µL of PBS. Secondary antibody (Alexa Fluor 488-conjugated goat anti-mouse IgG (Invitrogen, Carlsbad, CA); 1:500) was added to the plate at 50 µL/well. After incubation for 45 min at 37 °C, liquid was removed by aspiration and plates were washed three times with PBS. Approximately 40 µL of SlowFade™ Diamond Antifade Mountant with DAPI (Thermo Fisher Scientific, Waltham, USA) was added to each well to visualize nuclei of cells. The reactivity of the antibody and presence of nuclei, indicated by green (excitation at 488 nm, emission at 525 nm) and blue (excitation at 360 nm, emission at 460 nm) fluorescence, respectively, were observed using a fluorescence microscope (IX71, Olympus, Tokyo, Japan). In experiments shown in Supplementary Figs S2 and S3, we used inverted microscope Axio Observer Z.1 (Carl Zeiss, Oberkochen, Germany). Alexa Fluor 488 signals of each photo were quantified by NIS-Elements software (Nikon Corporation, Tokyo, Japan) and were expressed as a mean intensity per pixel.

### Pseudovirus neutralization test

To assess the neutralizing activity of CK47 against CHIKV envelope proteins, we performed a neutralization assay using CHIKV-pseudotyped lentiviral vector, as described previously^26^, though with some modification. Four-fold serial dilutions of CK47 (0.7 mg/ml), ranging from 1:200 to 1:204800, were mixed with CHIKV envelope protein-pseudotyped lentivirus and incubated at room temperature for 2 h. Mixtures of pseudotyped lentivirus and CK47 then were inoculated into cultures of U251 cells and maintained at 37 °C in a 5% CO_2_ environment. Twenty-four hours later, the medium was replaced with fresh MEM containing 2% FBS (v/v). At 72 hours post-infection, the cells in 100 µL of culture medium were lysed by mixing with an equal volume of substrate (Bright-Glo^TM^ Luciferase assay system, Promega Madison, WI) and the mixtures were incubated in the dark at room temperature for 10 min. The reactions were transferred into polystyrol microplates (Greiner Bio-one) and luciferase activities were measured. Relative luciferase units (RLU) were analyzed using a Synergy™ H1 microplate reader with Gen5™ Imager software (Biotek, Winooski, VT). Neutralization of pseudotyped lentivirus by CK47 was calculated as the percentage of infection of pseudotyped lentivirus using the following equation: (RLU of culture supernatant of cells treated with pseudotyped lentivirus and CK47 mixture/RLU of cells treated with pesudotyped lentivirus alone) × 100. Probit analysis was used to estimate the value of the CK47 dilution that showed inhibition of half of the maximum infection (EC_50_).

### Amino acid sequences and phylogenetic analysis

The amino acid sequences of CHIKV 6K-E1 proteins from viruses of the three genotypes isolated during various outbreaks were retrieved from the National Center for Biotechnology Information (NCBI) database. To analyze amino acid variation among genotypes, multiple sequence alignment was performed using the Molecular Evolutionary Genetic Analysis (MEGA) software, version 7.0^27^. The construction of a tree indicating the evolutionary relationship among the strains was performed using the nearest-neighbor-joining method with 1,000 replicate bootstraps.

### Statistical analysis

Data are presented as mean ± standard error of the mean (SEM). The unpaired two-tailed Student’s t test was used to compare data among 2 groups. Significance was determined at *P*-value lower than 0.05. All statistical analyses were performed using IBM SPSS software, version 20 (Armonk, NY:IBM Corp).

### Data availability

The datasets generated during the current study are available from the corresponding author on reasonable request.

## Electronic supplementary material

Supplementary Information
