# Supplementary material for: Variation at position 350 in the Chikungunya virus 6K-E1 protein determines the sensitivity of detection in a rapid E1-antigen test
Source: Sci Rep. 2018 Jan 18;8:1094. doi: 10.1038/s41598-018-19174-8 (PMC5773492; doi:10.1038/s41598-018-19174-8)
Supplement: Supplementary file 1 — Supplementary Information [file 41598_2018_19174_MOESM1_ESM.pdf]

Supplementary Information
Article in Scientific Reports

Variation at position 350 in the Chikungunya virus 6K-E1 protein determines the sensitivity of detection in a rapid E1-antigen test

Aekkachai Tuekprakhon[1,2], Emi E. Nakayama[2,3¶], Koen Bartholomeeusen[4¶], Orapim Puiprom[2], Tadahiro Sasaki[2], Ralph Huits[5], Natthanej Luplertlop[1], Nathamon Kosoltanapiwat[1], Pannamas Maneekan[6], Kevin K. Ariën[4,7], Tatsuo Shioda[2,3*], and Pornsawan Leaungwutiwong[1*].

[1] Department of Microbiology and Immunology, Faculty of Tropical Medicine, Mahidol University, Bangkok, Thailand
[2] Mahidol-Osaka Center for Infectious Diseases (MOCID), Faculty of Tropical Medicine, Mahidol University, Bangkok, Thailand
[3] Research Institute for Microbial Diseases (RIMD), Osaka University, Osaka, Japan
[4] Department of Biomedical Sciences, Institute of Tropical Medicine, Antwerp, Belgium
[5] Department of Clinical Sciences, Institute of Tropical Medicine, Antwerp, Belgium
[6] Department of Tropical Hygiene, Faculty of Tropical Medicine, Mahidol University, Bangkok, Thailand
[7] Department of Biomedical Sciences, University of Antwerp, Antwerp, Belgium
Variation at position 350 in the Chikungunya virus 6K-E1 protein determines the sensitivity of detection in a rapid E1-antigen test

[¶]These authors contributed equally to this work.
[*]Corresponding authors: shioda@biken.osaka-u.ac.jp (TS) and pornsawan.lea@mahidol.ac.th (PL)

Supplementary Fig S1

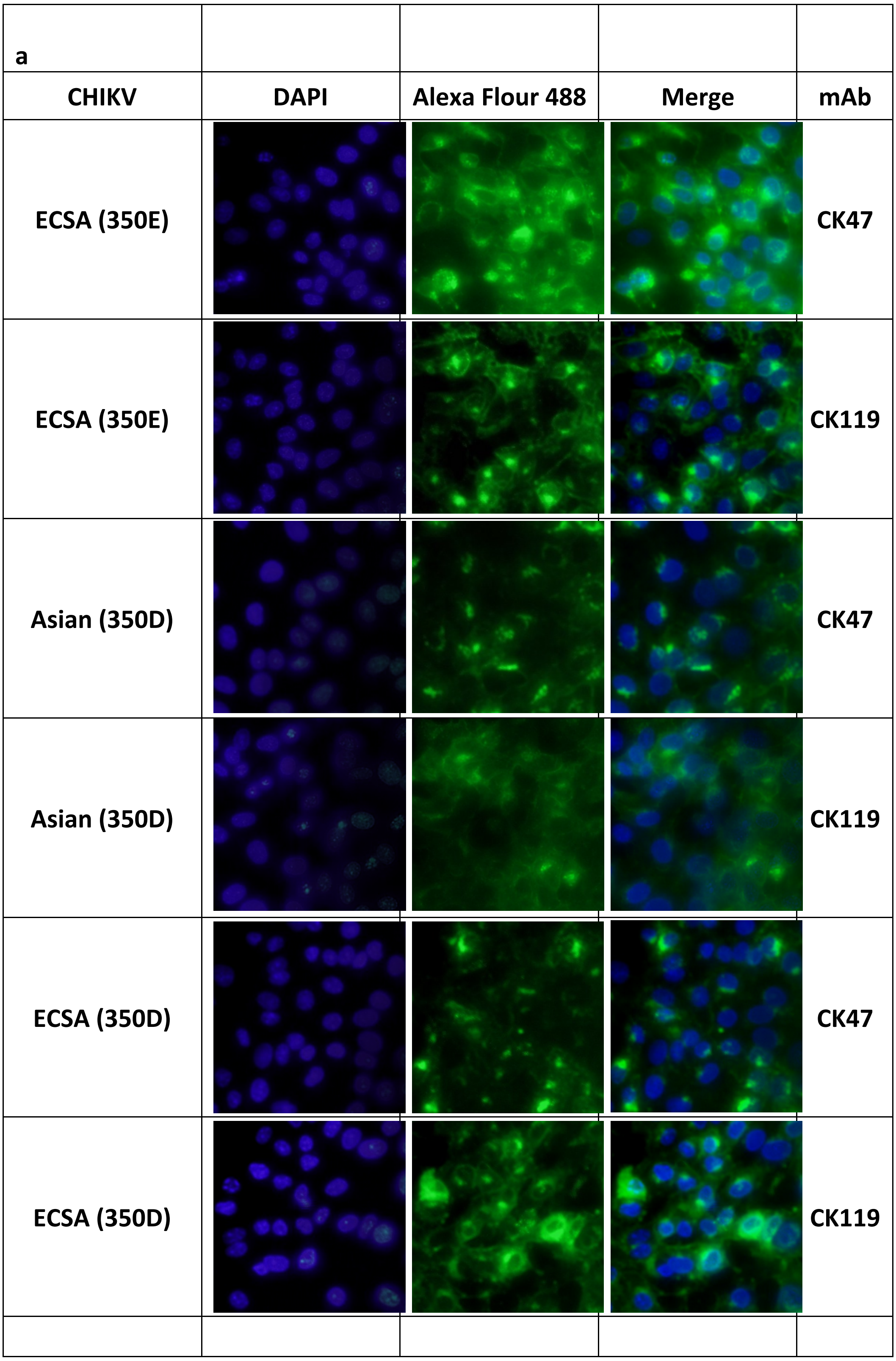

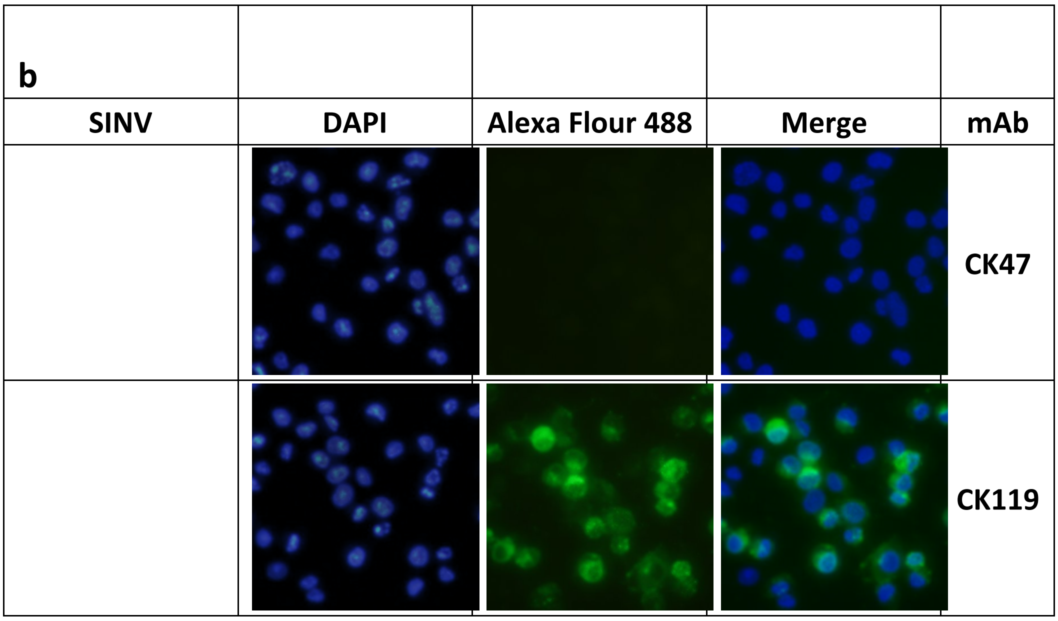

Supplementary Fig S1. Indirect immunofluorescence analysis of CK47 and CK119 labeling of virus-infected cells. CP10 (ECSA (350E)), ARUBA-15801125 (Asian (350D)), and Ross Low psg strain (ECSA (350D)) Chikungunya virus (CHIKV) -infected Vero cells **(a)** and Sindbis virus (SINV) -infected BHK cells **(b)** were stained with anti-CHIKV E protein monoclonal antibodies (mAb), CK47 or CK119; labeling was detected with Alexa Fluor 488-conjugated secondary antibody (green, middle). DAPI nuclear counterstain was used to stain nuclei of cells (blue, left). Alexa Fluor 488 and DAPI images were merged by ImageJ 1.5Oi, National Institutes of Health USA, and the merged images are shown on the right (Merge). Images represent results obtained from two independent experiments and were taken under 20X objective magnification using a fluorescence microscope (IX71, Olympus, Tokyo, Japan).

Supplementary Fig S2

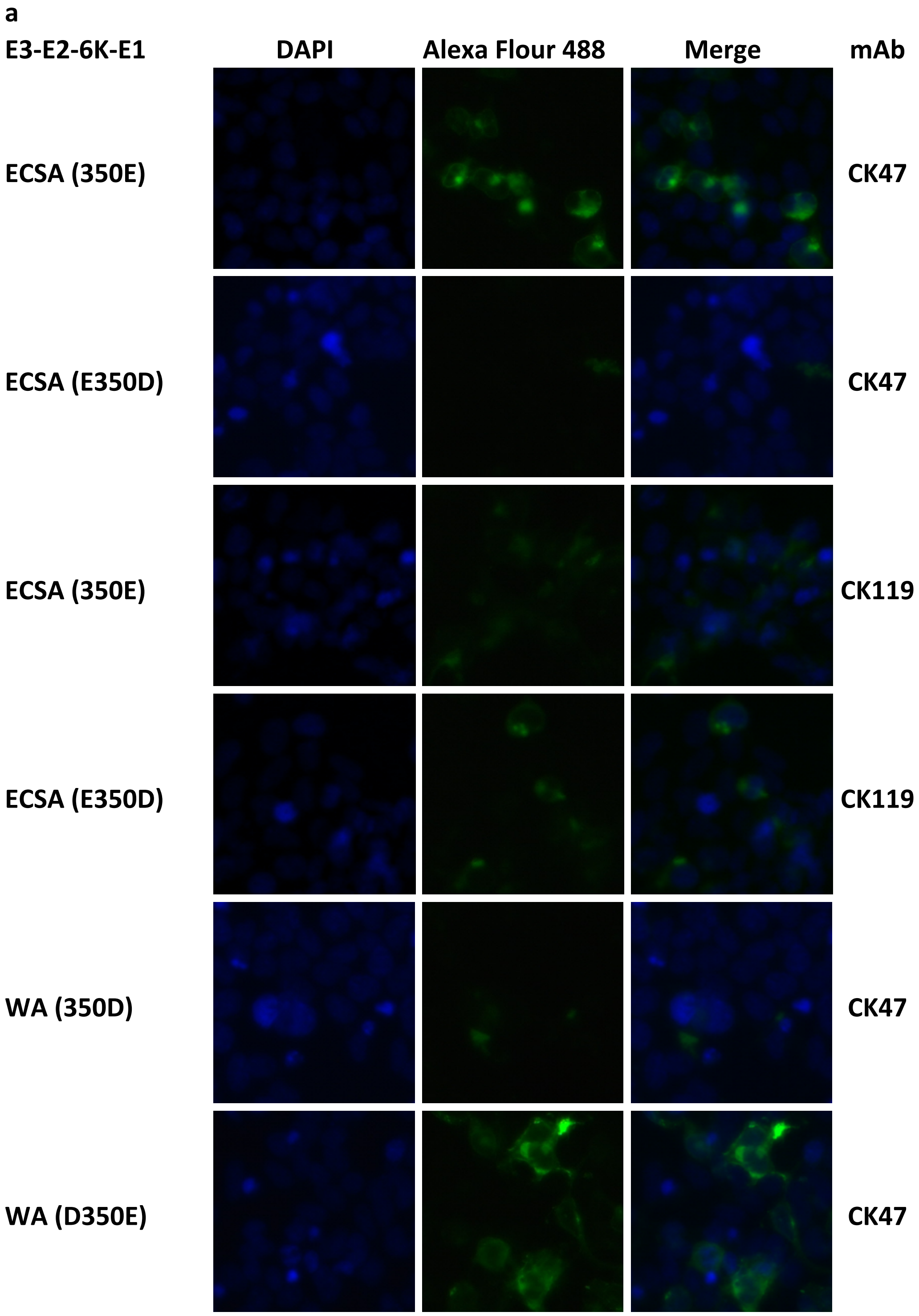

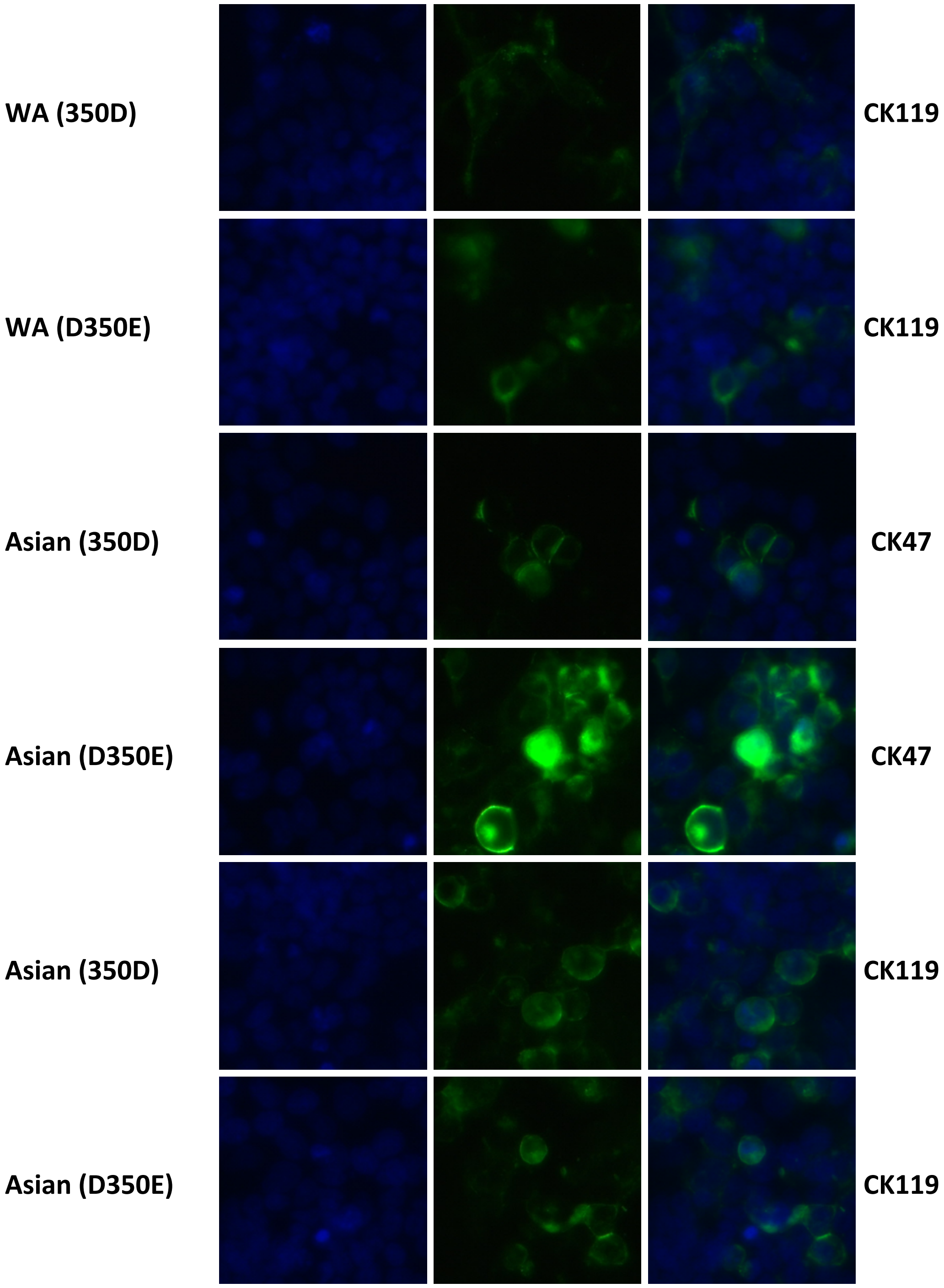
WA (350D)
CK119
WA (D350E)
CK119
Asian (350D)
CK47
Asian (D350E)
CK47
Asian (350D)
CK119
Asian (D350E)
CK119

b

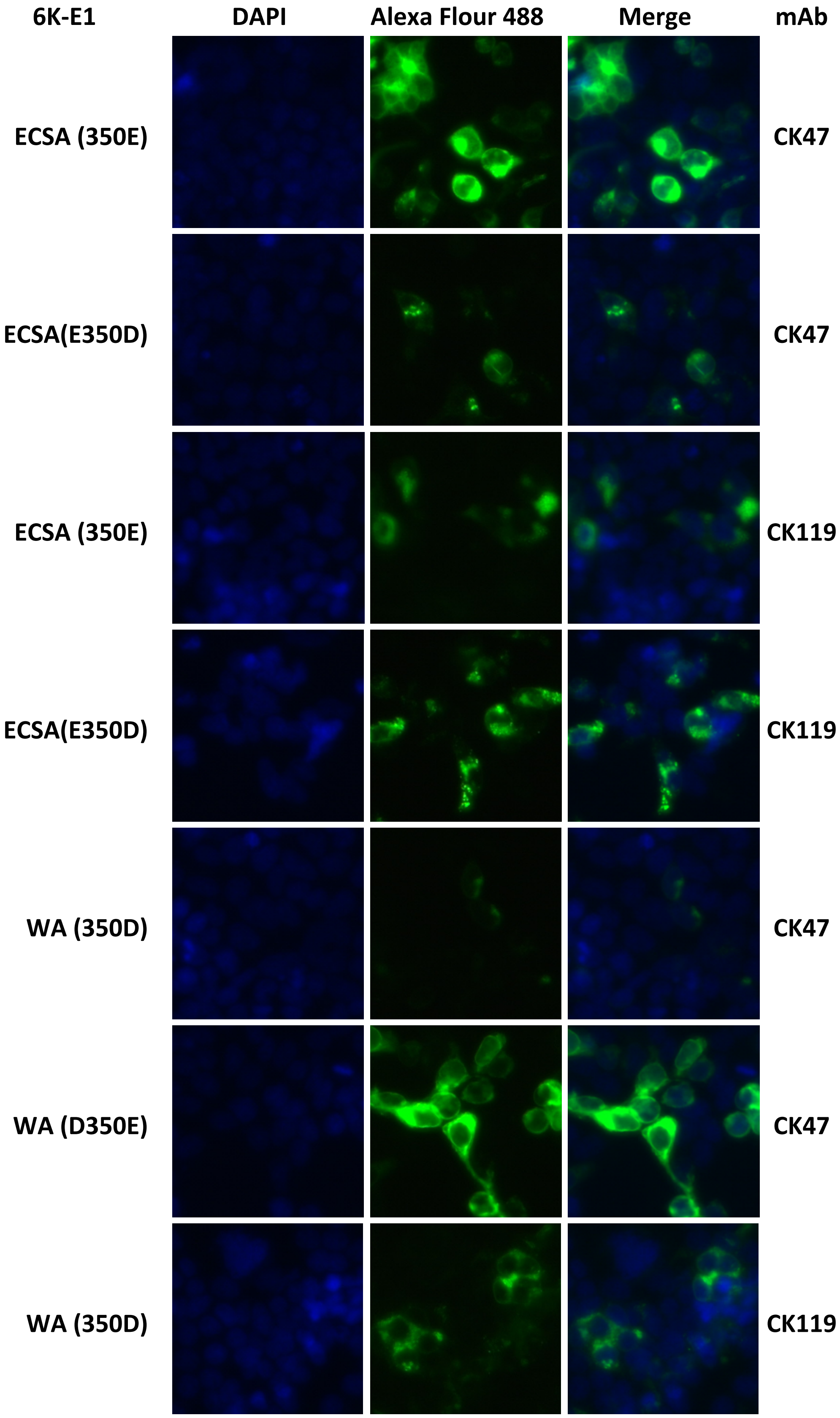
6K-E1
DAPI
Alexa Flour 488
Merge
mAb
ECSA (350E)
CK47
ECSA(E350D)
CK47
ECSA (350E)
CK119
ECSA(E350D)
CK119
WA (350D)
CK47
WA (D350E)
CK47
WA (350D)
CK119

WA (D350E)

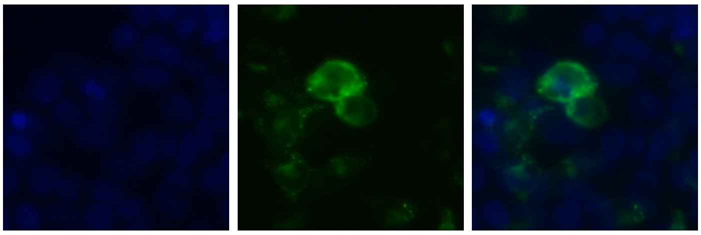

CK119

Supplementary Fig S2. Indirect immunofluorescence analysis of CK47 and CK119 against CHIKV envelope proteins. Plasmids were constructed to encode the full-length CHIKV envelope protein (E3-E2-6K-E1) from the CP10 (ECSA), 37997 (WA), or CK12-686 (Asian) strains, or mutant envelope proteins from CP10 bearing E350D, 37997 bearing D350E, or CK12-686 bearing D350E **(a).** Plasmids encoding the short versions of the envelope protein (6K-E1) from the CP10 strain (ECSA) or 37997 (WA) strain bearing either the wild type or mutant residue at position 350 were also constructed **(b).** Plasmids encoding CHIKV envelope protein were transfected into HEK293T cells and Indirect Immunofluoresecence test was performed using CK47 and CK119. Alexa Fluor 488-conjugated secondary antibody was used to detect the expression of CHIKV envelope protein of transfected cells (middle, green). The counterstain DAPI was used to stain nuclei of HEK293T cells (left, blue). Alexa Fluor 488 and DAPI images were merged by ImageJ 1.5Oi, National Institutes of Health USA, and the merged images are shown on the right (Merge). Images are representative of results obtained from three independent experiments and were taken under 20x objective magnification using inverted microscope Axio observer Z.1, Carl Zeiss.

Supplementary Fig S3

Amino acid at position 292 and 350	DAPI	Alexa Flour 488	Merge	mAb
292V, 350E	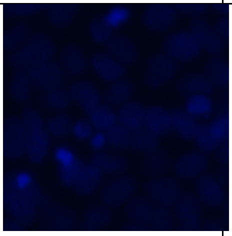	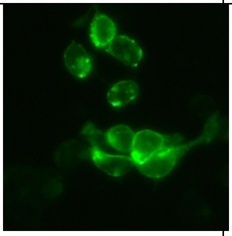	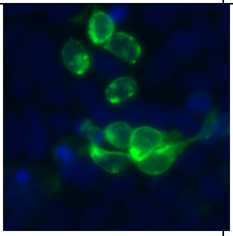	47
V292A, 350E	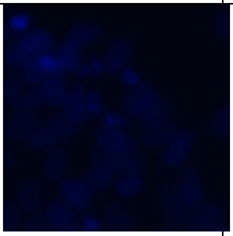	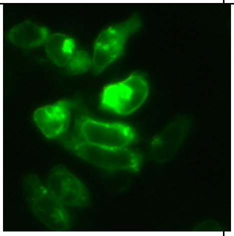	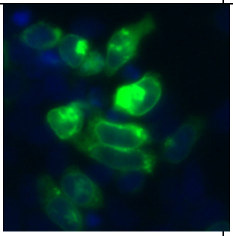	47
292V, E350D	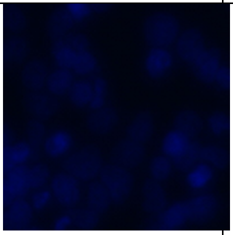	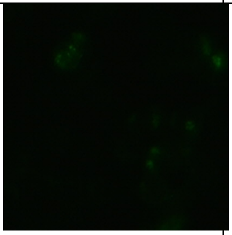	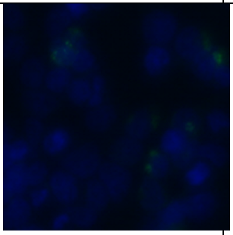	47
V292A, E350D	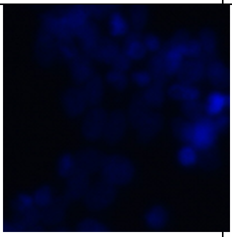	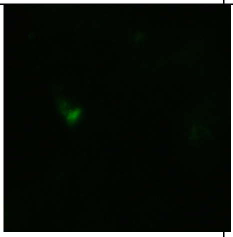	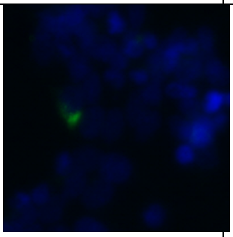	47
292V, 350E	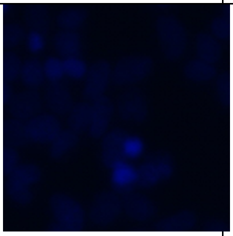	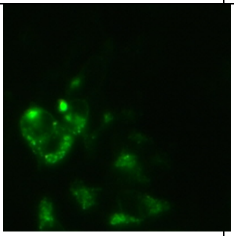	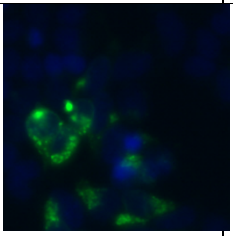	119
V292A, 350E	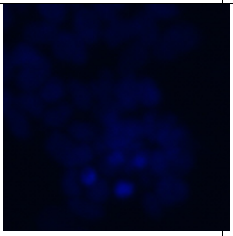	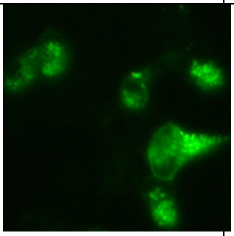	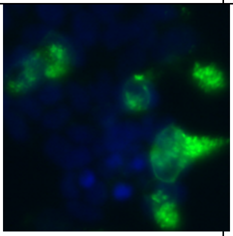	119
292V, E350D	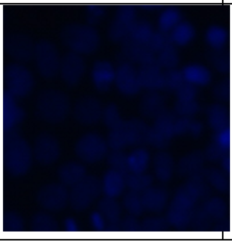	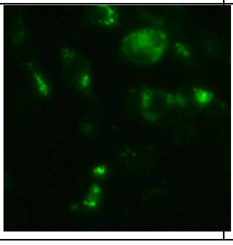	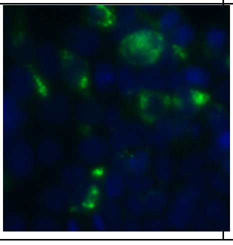	119

**V292A, E350D**	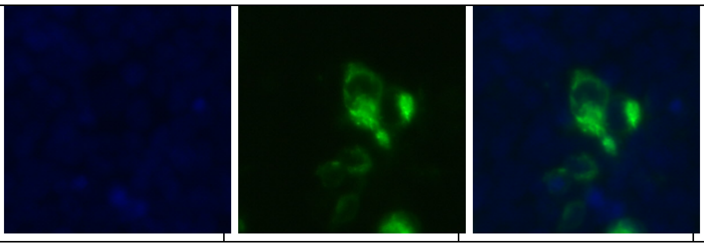	**119**

Supplementary Fig S3. Indirect immunofluorescence analysis of CK47 and CK119 against CHIKV envelope proteins. Site-directed mutagenesis was used to introduce mutations encoding substitutions at positions encoding residues 292 and 350 of 6K-E1 from the CP10 strain. Four possible combinations of 292 and 350 amino acid positions were produced: 292V + 350E, 292V + E350D, V292A + 350E, and V292A + E350D. Immunofluorescent images display reactivity of CK47 and CK119 against proteins of each of these four sequences. Alexa Fluor 488-conjugated secondary antibody was used to detect the expression of CHIKV envelope protein of transfected cells (middle, green) while DAPI nuclear counterstain was used to stain nuclei of HEK293T cells (left, blue). Alexa Fluor 488 and DAPI images were merged by ImageJ 1.5Oi, National Institutes of Health USA, and the merged images are shown on the right (Merge). Images are representative of results obtained from two independent experiments and were taken under 20x objective magnification using inverted microscope Axio observer Z.1, Carl Zeiss.

Supplementary Table S1. Details of CHIKV strains in the phylogenetic tree in Fig 3a.

Country	Year	Strain	Accession Number
CHINA	2010	GD05	JX088705.1
CHINA	2010	GD113	HQ846357.1
FRANCE LA REUNION	2005	IMTSSA6424C	CBX54834.1
THAILAND	2010	CP9	AB857809.1
THAILAND	2010	CP11	AB857825.1
THAILAND	2009	CU-Chik683	GU301781.1
SINGAPORE	2008	SGEHICHT077808	FJ445484.2
MALAYSIA	2008	0812bTw	FJ807893.1
CAMBODIA	2011	V1024314 KH11 PVH	JQ861258.1
MALAYSIA	2009	0901aTw	FJ807895.1
SRI LANKA	2008	LK(PB)chik6008	GU013529.2
THAILAND	2010	CP1	AB857793.1
THAILAND	2010	CP13	AB857833.1
THAILAND	2010	CP16	AB857841.1
MAURITIUS	2006	D570/06	ABJ98544.1
CHINA	2010	DG892	JQ065886.1
SINGAPORE	2008	SGEHICHS424108	FJ445443.2
THAILAND	2008	CU-Chik10	GU301780.1
THAILAND	2010	CP10	AB857817.1
THAILAND	2010	CP7	AB857801.1
FRANCE LA REUNION	2006	LR2006-OPY1	ABD95938.1
INDIA	2006	IND-06-MH2	EF027136.1
SRI LANKA	2007	SL-CK1	HM045801.1
SRILANKA	2009	SL111314	AB455493.1
CENTRAL AFRICA REPUBLIC	1984	DakAr B1	HM045784.1
CENTRAL AFRICA REPUBLIC	1978	HB78	HM045822.1
UGANDA	1982	UgAg4155	HM045812.1
BRAZIL	2014	H804705	KP164569.1
BRAZIL	2016	CHIKVgp2	ANK58565.1
BRAZIL	2014	BHI3734 H804698	KP164568.1
BRAZIL	2014	BHI3745 H804709	KP164570.1
CENTRAL AFRICA REPUBLIC		CAR256	HM045793.1
CONGO	1960	LSFS	HM045809.1
SOUTH AFRICA	1976	AR18211	HM045805.1
SOUTH AFRICA	1976	SAH2123	HM045795.1
TANZANIA	1953	Ross Low psg	HM045811.1
TANZANIA	1952	S27	NC004162
SOUTH AFRICA	1956	Vereeniging	HM045792.1
INDIA	1963	I-634029	HM045803.1
INDIA	1973	PO731460	HM045788.1
INDIA	1963	Gibbs 63-263	HM045813.1
THAILAND	1978	3412-78	HM045808.1

THAILAND	1988	6441-88	HM045789.1
THAILAND	1995	CO392-95	HM045796.1
THAILAND	1995	K0146-95	HM045802.1
THAILAND	1995	SV0444-95	HM045787.1
INDONESIA	1983	JKT23574	HM045791.1
PHILIPPINES	1985	Hu-85-NR-001	HM045800.1
INDONESIA	1985	RSU1	HM045797.1
PHILIPPINES	1985	PhH15483	HM045790.1
THAILAND	1975	1455-75	HM045814.1
BRAZIL	2014	PER160 H803609	KP164571.1
BRAZIL	2014	TR206 H804187	KP164572.1
CARIBIAN	2014	G105	LN898103.1
COLUMBIA	2016	COL.UF-1	KX496989.1
PUERTO RICO	2014	PR-S6	KR264951.1
ARUBA	2016	ARUBA-15800567	
ARUBA	2016	ARUBA-15801125	MF682981
ARUBA	2016	ARUBA-15801654	MF682985
ARUBA	2016	ARUBA-15802650	
PHILIPPINES	2012	CK12-686	CWIH00000000.1
PHILIPPINES	2012	CK12-921	CXNT00000000.1
PUERTO RICO	2014	PR-S4	KR264949.1
PHILIPPINES	2012	CK12-559	WHX00000000.1
ARUBA	2016	ARUBA-15801136	MF682982
ARUBA	2016	ARUBA-15801160	MF682983
ARUBA	2016	ARUBA-15801358	MF682984
COTE D IVOIRE	1981	ArA 2657	HM045818.1
SENEGAL	2005	HD 180760	HM045817.1
COTE D IVOIRE	1993	ArA 30548	HM045820.1
NIGERIA	1964	IbH35	HM045786.1
NIGERIA	1965	IbAn4824	HM045807.1
SENEGAL	1983	37997	AY726732.1
SENEGAL	1966	PM2951	HM045785.1
SENEGAL	1966	SH2830	HM045798.1
SENEGAL	1966	SH 3013	HM045816.1
SENEGAL	1979	ArD 30237	HM045815.1
